# RNA Interference Therapies for an HIV-1 Functional Cure

**DOI:** 10.3390/v10010008

**Published:** 2017-12-27

**Authors:** Robert J. Scarborough, Anne Gatignol

**Affiliations:** 1 Lady Davis Institute for Medical Research, Montreal, QC H3T 1E2, Canada; anne.gatignol@mcgill.ca; 2 Department of Microbiology and Immunology, McGill University, Montreal, QC H3A0G4, Canada; 3 Department of Medicine, Division of Experimental Medicine, McGill University, Montreal, QC H3A0G4, Canada

**Keywords:** HIV-1, functional cure, cell transplant, RNA interference, small/short interfering RNA, small/short hairpin RNA, micro RNA

## Abstract

HIV-1 drug therapies can prevent disease progression but cannot eliminate HIV-1 viruses from an infected individual. While there is hope that elimination of HIV-1 can be achieved, several approaches to reach a functional cure (control of HIV-1 replication in the absence of drug therapy) are also under investigation. One of these approaches is the transplant of HIV-1 resistant cells expressing anti-HIV-1 RNAs, proteins or peptides. Small RNAs that use RNA interference pathways to target HIV-1 replication have emerged as competitive candidates for cell transplant therapy and have been included in all gene combinations that have so far entered clinical trials. Here, we review RNA interference pathways in mammalian cells and the design of therapeutic small RNAs that use these pathways to target pathogenic RNA sequences. Studies that have been performed to identify anti-HIV-1 RNA interference therapeutics are also reviewed and perspectives on their use in combination gene therapy to functionally cure HIV-1 infection are provided.

## 1. Introduction

Several drugs are available for combination antiretroviral therapy (cART), the current standard of care for HIV-1 infection. They target the HIV-1 enzymes reverse transcriptase, protease and integrase as well as the cellular entry receptors, CD4 and C-C chemokine receptor type 5 (CCR5) [1,2,3]. Soon after the first effective cART regimens became available, it was shown that HIV-1 persists in drug resistant reservoirs, even after prolonged therapy [4]. Resting CD4^+^ T cells, that can harbor latent infectious HIV-1 genomes, were identified as a major reservoir for HIV-1 [5] and it has been estimated that it would take over 60 years to clear HIV-1 from that reservoir with cART [6]. Attempts to eliminate reservoirs have so far been unsuccessful [7,8,9] and alternative approaches to cure HIV-1 infection are under investigation. A functional cure can be defined as an intervention that leads to control of HIV-1 replication in the absence of cART, without necessarily eliminating the virus from reservoirs. Ideally, a functional cure would be permanent; however, long-term remissions from drug therapy may also be considered as a kind of temporary functional cure. Long-term remissions have been observed in several individuals who initiated cART early following infection. Documented cases include the Mississippi baby [10,11], a young woman from the French Agence Nationale de Recherche sur le Sida (ANRS) EPFCO10 pediatric cohort and several patients from the ANRS VISCONTI (Viro-Immunologic Sustained Control after Treatment Interruption) study [12,13,14]. While these cases show that early treatment can result in at least a temporary functional cure, initiating early treatment cannot help those already infected with HIV-1 and most people who will become infected won’t be diagnosed soon enough for this approach to be successful. Other approaches to attain a functional cure include pharmacologically inducing a deep latency of HIV-1 replication [15,16], modulating the immune system to control HIV-1 replication [17], and transplanting HIV-1 resistant cells [18].

So far, one person has attained what appears to be a permanent functional cure through HIV-1 resistant cell transplant. Timothy Brown, also known as the Berlin patient, received an allogeneic hematopoietic stem cell (HSC) transplant from an individual with the homozygous CCR5∆32/∆32 deletion, a genotype that renders cells resistant to infection by HIV-1 R5 tropic viruses [19,20]. The treatment was prescribed to treat both relapsed acute myeloid leukemia and, potentially, HIV-1 infection [19]. Since his transplant in 2007, Timothy Brown has been off cART and remains the only individual with an established infection who has been functionally cured of HIV-1. Unfortunately, the high risk associated with allogeneic HSC transplant and the low incidence of known CCR5∆32/∆32 carriers makes the protocol used in his case inaccessible to the vast majority of HIV-1-infected individuals.

The idea of using cell transplant to treat HIV-1 infection had been proposed early in the HIV-1 epidemic (1988) [21]. The first clinical trials were completed in the late 1990s using autologous transplant of CD4^+^ T cells transduced ex vivo with a gene directing the production of an antiviral protein [22,23] or RNA [24,25,26]. Since then, several clinical trials have been conducted to evaluate the safety and efficacy of diverse protein-based and RNA-based genes, alone and in combination (summarized in Table 1).

Protein-based genes that have advanced to clinical trials include trans-dominant HIV-1 Rev proteins that inhibit the export of singly-spliced and full-length HIV-1 RNA to the cytoplasm [22,23,27,28,29,30], a gp41 C peptide (C46) that inhibits viral entry [31] and a human-rhesus tripartite motif 5 α chimeric protein (TRIM5α-HRH) that targets the HIV-1 capsid [41,42]. A major concern for use of these genes is that they may produce peptides that are recognized by adaptive immune responses as foreign, which could result in chronic immune activation and/or clearance of HIV-1 resistant cells [43]. Another protein-based approach for cell transplant therapy is the transient delivery of gene editing enzymes to modify the CCR5 gene and phenotypically mimic the CCR5∆32/∆32 genotype of the donor used for the Berlin patient. Transient delivery of both a Zinc finger nuclease [32] and, more recently, a guide RNA/Cas9 (Clustered Regularly Interspaced Short Palindromic Repeats (CRISPR) associated 9 protein) system [44], are being used to modify the CCR5 gene in ongoing clinical studies (Table 1). While it is anticipated that these approaches will be effective, the long-term safety of these enzymes has not yet been evaluated and modifying the CCR5 gene would only be effective for people infected exclusively with viruses that use CCR5 as a co-receptor for entry into cells.

Because of their small size and low potential to elicit adaptive immune responses, several anti-HIV-1 RNAs have advanced to clinical trials. They can be grouped into those that act through antisense-based mechanisms to target RNA and those that act through decoy or aptamer mechanisms to target proteins. Decoy RNAs have been designed to mimic the transactivation response (TAR) and Rev response element (RRE) structures in HIV-1 RNA and inhibit the HIV-1 regulatory proteins Tat and Rev, respectively [45]. Both RRE and TAR decoys have entered clinical trials (Table 1); however, their ability to saturate components of the RNA interference (RNAi) machinery has been described as a potential limitation for their use in combination therapy [46,47]. RNA aptamers identified using systematic evolution of ligands by exponential enrichment (SELEX) have also been selected to target the HIV-1 proteins RT [48,49], protease [50], integrase [51] and the Gag polyprotein [52].

Antisense-based RNAs all act through specific base pairing with an RNA target sequence. Those that have entered clinical trials include single-stranded antisense RNAs, that can block RNA translation, interfere with RNA processing or recruit RNA enzymes (RNases) to degrade their target RNA (reviewed in [53]), ribozymes, that can bind to and cleave a target RNA sequence (reviewed in [54]) and short hairpin RNAs (shRNAs) that use the cellular RNA interference (RNAi) machinery to cleave their target RNAs. shRNAs have been shown to have 50% inhibitory values of about 100-fold less compared to target matched ribozymes and antisense RNAs [55]. They have emerged as some of the top candidates for anti-HIV-1 cell transplant therapy and have been included in all combinations that have so far entered clinical trials (Table 1). Here we review the RNAi machinery, RNA therapeutics that use the RNAi machinery and RNAi therapies that have been designed for use in cell transplant therapy to functionally cure HIV-1 infection.

## 2. RNA Interference (RNAi) Pathways

### 2.1. RNAi Defense Mechanism

**Figure 1:**
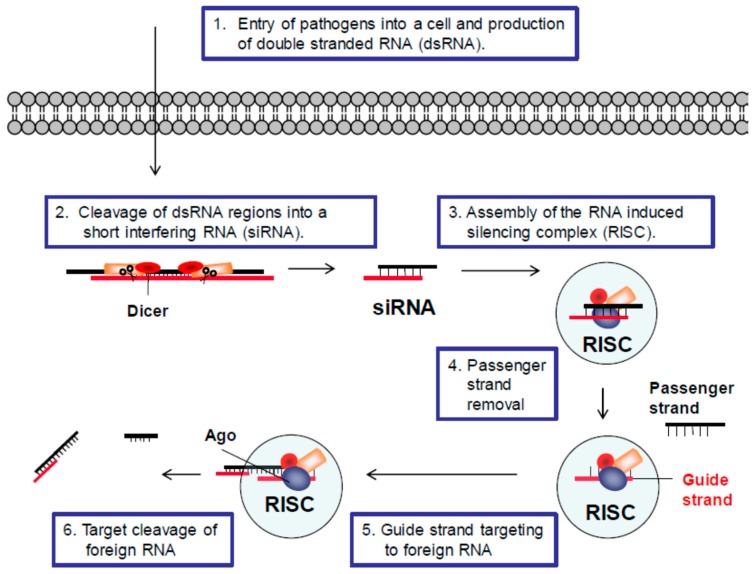
**RNA interference (RNAi) defense pathway.** The general steps of the RNAi defense pathway are illustrated: (1) Double stranded RNAs (dsRNA) are produced from pathogens such as viruses, satellite RNAs and retrotransposons. (2) Regions of pathogenic dsRNAs are recognized by a complex including the Dicer enzyme, which processes these regions into small interfering RNAs (siRNA). (3) siRNAs are then loaded into the RNA-induced silencing complex (RISC). (4) The passenger strand is removed from the RISC. (5) The guide strand directs the RISC to its complementary target in the pathogenic RNA. (6) The target sequence is cleaved by an Argonaute (Ago) protein in the RISC.

RNAi was first described in 1998 using cells from *Caenorhabditis elegans* (*C. elegans*) [56]. Nearly all eukaryotic cells can use the RNAi pathway to target foreign double stranded RNA, and in many organisms it is a major defense mechanism against pathogens [57]. Defense is accomplished through the processing of double stranded RNA (dsRNA) regions of pathogenic RNA into small or short interfering RNAs (siRNAs). siRNAs then associate with cellular proteins to form an RNA-induced silencing complex (RISC), which can cleave the sequence of foreign RNA from which the siRNA was derived. A general illustration of this process is provided in Figure 1. In most cells, the RNase responsible for generating the siRNAs is called Dicer and the RNase responsible for cleaving the target RNA is an Argonaute (Ago) protein [58].

Although well defined as a defense mechanism in plants and invertebrates [57], the role of RNAi in defense against mammalian pathogens has been debated [59,60,61]. In mammalian embryonic stem cells, that do not have a functional interferon system, RNAi defense was observed [62,63,64] and defense against viruses deleted for proteins acting as RNAi suppressors [65] or when the interferon pathway is inactivated [66] has also been observed. Given the variety of protein-based RNA immune sensors available in mammalian cells, RNAi immunity may be restricted to certain cell types and pathogens. While it is clear that the si/sh RNAi pathway is functional in human cells [58], additional studies are needed to determine what role it plays in defense against diverse human pathogens.

### 2.2. RNAi Post-Transcriptional Gene Regulation Mechanism

**Figure 2:**
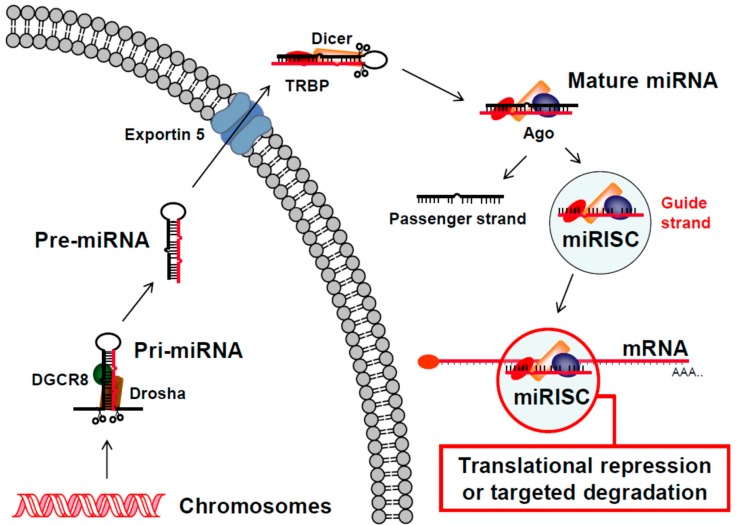
**Human micro RNA (miRNA) pathway.** The general steps of the human miRNA pathway are illustrated starting with expression of a primary miRNA (Pri-miRNA) from a cell’s chromosomes (bottom, left). The Pri-miRNA is recognized by the endonuclease Drosha in complex with the RNA binding protein DiGeorge syndrome chromosomal region 8 (DGCR8). Drosha cleaves the Pri-miRNA into a precursor miRNA (Pre-miRNA) with a 3′ overhang. The Pre-miRNA is then exported to the cytoplasm by the Exportin 5 transporter complex and recognized by the endonuclease Dicer in complex with the Trans-activation response RNA binding protein (TRBP). Dicer cleaves the loop off of the Pre-miRNA to generate a mature miRNA. Following recruitment of additional proteins, including an Argonaute (Ago) protein, the passenger strand of the miRNA is removed and the miRISC complex targets complementary sequences in mRNA transcripts for translational repression or targeted degradation (bottom, right).

In 2001, a role for the RNAi pathway in post-transcriptional gene regulation mediated by small dsRNAs called microRNAs (miRNAs) was first described using cells from *C. elegans* [67,68,69] and it has since been characterized in nearly all eukaryotic organisms. Most mammalian genes are targets of this regulation [70] and as of 2014 thousands of mature human miRNA sequences had been deposited in the miRBase database (http://www.mirbase.org/) with 278 annotated with high confidence [71]. Like siRNAs (Figure 1), miRNAs use the RNAi pathway to target sequences in RNA that are complementary to their guide strand. In human cells, they are derived from primary (pri-) miRNAs, which are expressed predominantly from their own RNA Polymerase (Pol) II promoters, but can also be found in introns of other genes or expressed from RNA Pol III promoters [72]. Pri-miRNAs form a hairpin structure, which is cleaved by the RNase Drosha in complex with the RNA binding protein DiGeorge syndrome chromosomal region 8 (DGCR8), to produce a 60 to 80 nucleotide precursor (pre-) miRNA with a two-nucleotide 3′ overhang at the base of the hairpin stem (Figure 2). Nuclear export occurs via Exportin 5 [73] and once in the cytoplasm, pre-miRNAs form a complex with the RNase Dicer and the Trans-activation response RNA binding protein (TRBP) [74]. This complex directs the cleavage of the hairpin loop to produce a mature miRNA with a two-nucleotide 3′ overhang on each strand. An Argonaute (Ago) protein is then recruited to form the miRNA RISC (miRISC). Because of their imperfect complementarity with their targets in different messenger RNAs (mRNAs), miRNAs typically direct translational repression or targeted degradation (Figure 2), as opposed to targeted cleavage directed by siRNAs (Figure 1).

In addition to their role in cellular gene regulation, both viral and cellular derived miRNAs have been described as playing important roles in the regulation of mammalian viruses (reviewed in [75]). For example, Ouellet et al. identified two functional miRNAs derived from the HIV-1 TAR element [76] and Huang et al. demonstrated that several cellular miRNAs contribute to HIV-1 latency in resting CD4^+^ T cells [77]. As models of RNAi evolve and new pathways are identified, unraveling the intricacies of its role in immunity and gene regulation will remain an important focus of research for several years to come. Our understanding of these mechanisms has also provided several new classes of potential therapeutic molecules that use the RNAi pathway to target pathogenic RNAs or modify pathogenic gene expression.

## 3. RNAi Therapeutics

Improvements in the chemistry of and delivery vehicles for therapeutic siRNAs have helped overcome several barriers to their clinical development and there are a number of siRNAs in early and late phase clinical trials for a variety of human afflictions (reviewed in [78]). While not yet an approved form of medication, Anylam Pharmaceuticals has several siRNAs in late stage clinical development with positive phase III results recently reported for their siRNA, patisiran (NCT01960348), for treatment of transthyretin amyloidosis [79]. siRNAs can be designed to decrease the expression of any protein involved in human disease and they have the potential to be used as medicines to treat many disorders with no available therapies [78]. Although several setbacks have been encountered on their path to development, encouraging results from recent clinical studies suggest that the much anticipated explosion of siRNAs as medicines for human disease will soon become a reality. siRNAs can also be delivered from DNA templates that express shRNAs, sometimes referred to as DNA-directed RNAi. Although several different viral and non-viral vectors have been used for their delivery, adeno-associated viruses have emerged as top candidates for long-term expression in non-dividing cells (reviewed in [80]) and retroviral vectors can be used for permanent expression in dividing cells (reviewed in [81]). In the following sections, we review the development of siRNAs and shRNAs as well as the different formats that have been evaluated for their use as therapeutics.

### 3.1. Small/Short Interfering RNAs (siRNAs)

Although the delivery of dsRNA could elicit an RNAi response in cells from diverse organisms, a similar induction of mammalian RNAi could not initially be observed in several different cell lines because they activate the interferon pathway [82,83]. Following the identification of 21 to 23 nucleotide siRNAs as the processing products of dsRNA and effectors of the RNAi pathway in *Drosophila* [84,85] and *C. elegans* [86], Elbashir et al. and Caplen et al. showed that the delivery of these siRNAs to a variety of mammalian cell lines could elicit a potent RNAi response without activating the interferon pathway [87,88]. Results also suggested that the failure to observe effective inhibition using longer double stranded RNAs (>30 base pairs) was due to activation of immune responses, masking RNAi effects. These findings gave rise to the application of siRNAs as both research tools and potential therapeutic molecules.

**Figure 3:**
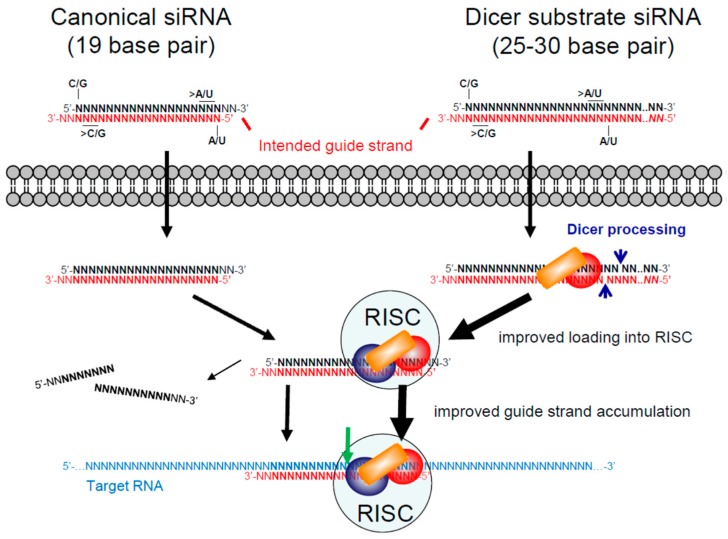
**Canonical and Dicer substrate siRNA designs.** On the top left, the typical or canonical siRNA design of 19 base pairs with two-nucleotide overhangs on the ends is illustrated. To ensure that the intended guide strand (red) is selected by the RNA induced silencing complex (RISC), siRNAs should have higher G/C content at the 3′ end of the intended guide strand and higher A/U content at its 5′ end, in particular for the terminal nucleotides. On the top right, Dicer substrate siRNA designs of 25 to 30 base pairs are illustrated. They have been designed both in symmetrical two-nucleotide overhang formats and with a 5′ blunt end (*NN*) on the intended guide strand. Both canonical and Dicer substrate siRNAs use the RISC to cleave their target RNA (green arrow). By first recruiting the Dicer enzyme complex, Dicer substrate siRNAs may improve loading of siRNAs into the RISC and improve preferential selection of the guide strand.

The most commonly used siRNA design consists of two 21 nucleotide RNA strands that form 19 base pairs with a two-nucleotide overhang at the 3′ end of each strand, often referred to as a canonical siRNA (Figure 3). In this format, the siRNA does not need to be cleaved by Dicer and can enter the RISC directly. The selection of the guide strand by the RISC depends on the thermodynamic stability of the ends [89,90] and designing siRNAs with lower thermodynamic stability at the 5′ end of the intended guide strand (>A/U content) and higher stability at the 5′ end of the intended passenger strand (>G/C content) ensures that the intended guide strand is preferentially selected [91]. Other sequence features, such as total G/C content and presence of immune stimulatory sequences, can be used to select siRNAs with high potential for efficacy and low potential for off-target effects (reviewed in [91]).

Results published in 2005 suggested that longer siRNAs, that must first be cleaved by Dicer, were more effective compared to their sequence-matched canonical siRNAs [92]. Dicer substrate siRNAs are commonly designed with a 25 nucleotide passenger strand and a 27 nucleotide guide strand that bind with a blunt end at the 5′ end of the intended guide strand (25/27) [93,94] (Figure 3). By recruiting the Dicer enzyme complex, it was suggested that these longer RNAs can more efficiently mediate RNAi [92,95]. It was subsequently shown that they improve RISC assembly, guide strand accumulation and guide strand recruitment of Ago proteins [95]. In 2012, a large-scale study reported no difference in the effects of target matched siRNAs and Dicer substrate RNAs [96]. Using a target site that our lab had identified in HIV-1 RNA [97], we evaluated the anti-HIV-1 production potency [98,99] of siRNAs with different lengths and symmetries [100]. While the typical Dicer substrate siRNA format (25/27) was much less potent compared to a canonical design (21/21), longer siRNAs (27 to 30 base pairs) with one blunt end or symmetrical two-nucleotide overhangs were several fold more potent. We speculated that the failure of the 2012 study to observe an advantage of Dicer substrate siRNA designs [96] was because the typical 25/27 format was not long enough for several target sites to benefit from recruitment of the Dicer complex. However, more studies are needed to conclude whether there is a real advantage of recruiting the Dicer complex and what the minimum length is to harness this advantage.

In addition to the Dicer substrate design, several other non-canonical siRNA formats have been evaluated, including one or two blunt ends, various strand lengths and different nucleotide modifications (reviewed in [101]). While there are conflicting results regarding the optimal length and format of therapeutic siRNAs, it is likely that the optimal design for any particular therapeutic target site will require empirical data to identify the safest and most efficacious molecule for development.

### 3.2. Small/Short Hairpin RNAs (shRNAs)

**Figure 4:**
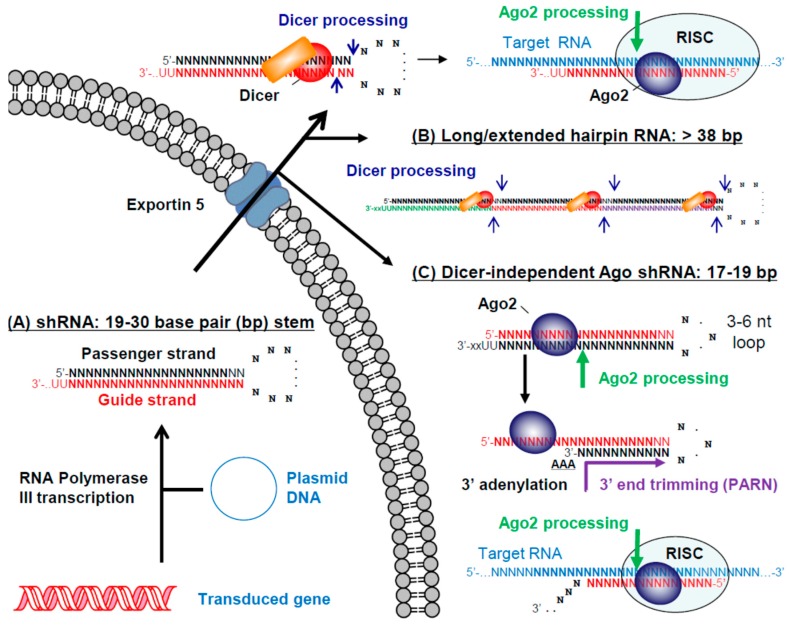
**Therapeutic short hairpin RNAs (shRNAs).** (**A**) shRNAs with 19 to 30 base pair (bp) stems and variable loop sequences can be transcribed from an integrated gene or transfected plasmid DNA using RNA polymerase III (Pol III) promoters. Transcription starts from the 5′ end of the intended passenger strand and terminates with two or more Us transcribed from the RNA Pol III termination signal (five or more As) at the 3′ end of the intended guide strand. The shRNA is transported out of the nucleus by Exportin 5, and the loop is cleaved off by the Dicer enzyme complex. Proteins of the RNA induced silencing complex (RISC) help direct Ago2 to cleave and remove the passenger strand and subsequently to cleave target RNA sequences complementary to the guide strand; (**B**) an example of a long or extended shRNA is illustrated. Sequential guide strands with the same or different targets can be incorporated with up to three active guide strands being generated by Dicer processing; (**C**) an Ago-shRNA design with a 17 to 19 bp stem and a 3 to 6 nucleotide loop is illustrated. Unlike standard shRNAs, the intended guide strand is located at the 5′ end of the transcript and the intended passenger strand is on the 3′ end. In this format the shRNA is too small to be cleaved by Dicer. Instead it is bound by Ago2, which cleaves the intended passenger strand. Although the details are not fully elucidated, the 3′ end is thought to be adenylated, followed by 3′ end trimming by the poly(A)-specific ribonuclease (PARN). The released guide strand can then direct Ago2 to cleave a target RNA. The RISC in this case may have a different composition compared to that used by a standard shRNA design.

Shortly after the first descriptions of effective gene silencing by synthetic siRNAs in mammalian cells, Brummelkamp et al. showed that siRNAs could also be delivered to mammalian cells from DNA vectors expressing short hairpin RNAs (shRNAs) [102]. shRNAs are typically expressed from type 3 RNA Pol III promoters such as human H1, U6 and 7SK. From these promoters transcription starts at the 5′ end of the intended passenger strand and terminates at the 3′ end of the intended guide strand, resulting in a guide strand overhang of two to four uridines [103] (Figure 4A). Recent results have shown that the transcription start [104,105] and end [106] sites of small RNAs expressed from type 3 RNA Pol III promoters can vary by a few nucleotides. To ensure that the highest percentage of intended transcripts are produced, different promoters should be compared for therapeutic shRNAs and additional research is needed to optimize promoter sequences for the DNA-directed delivery of small RNAs in general.

An shRNA transcript closely resembles a pre-miRNA except that it has perfect complementarity both between the passenger and guide strands in the shRNA stem and between the intended guide strand and its target RNA. As a result, shRNA guide strands can recruit Ago2 to cleave their target RNA, as opposed to most miRNA guide strands that bind with imperfect complementarity to their target sites and direct translational repression or targeted degradation. Like siRNAs, several alternative shRNA formats have been evaluated including alternative loop sequences and different stem lengths (reviewed in [103]). Although it had been suggested that both longer [107,108] and shorter [109] shRNAs are more effective formats, McIntyre et al. observed no correlation between stem length and suppressive activity for 91 shRNAs targeting HIV-1 RNA [110]. We compared siRNAs and shRNAs with different lengths targeting a site in HIV-1 RNA and found no correlation between the suppressive activity of siRNA and shRNA length variants [100]. Since shRNAs require Dicer to cut off their loop sequence, they do not need to be longer in order to recruit the Dicer complex. In our study, while the optimal siRNA lengths for a single target site were longer (27 to 29 base pairs), the optimal shRNA lengths were shorter (20 to 21 base pairs). Based on the available evidence, there is no way to predict the optimal length of a therapeutic shRNA and different lengths should be tested to ensure that the best format for a particular target site is advanced into clinical trials.

An alternative therapeutic design to deliver siRNAs from a DNA vector is the use of miRNA scaffolds, which include the tail region of a miRNA that must first be cleaved by Drosha. However, two studies concluded that these formats are less potent compared to target matched shRNAs that bypass this step [111,112]. While less potent, miRNA designs can be expressed from RNA Pol II promoters, which may be advantageous for certain therapeutic strategies, such as those requiring tissue specific expression from a cell-type specific promoter. The miRNA design has also been used to express several miRNAs in a single transcript, taking advantage of Drosha processing to release each individual miRNA [113,114,115]. For a similar purpose, long or extended hairpins have been designed [116,117], from which Dicer processing leads to the release of two or more siRNAs (Figure 4B).

Dicer-independent Ago shRNAs have also been designed based on the unique biogenesis pathway of miRNA 451 (miR-451) [118,119,120,121]. Unlike other miRNAs, the pre-miR-451 is processed by Ago2 instead of Dicer. To utilize miR-451 processing pathways, Ago shRNAs have been designed so that their intended guide strand is at the 5′ end of the hairpin and forms a 17 to 19 base pair stem with the intended passenger strand, connected by a short 3 to 6 nucleotide loop [122] (Figure 4C). In this format Ago2 can directly cleave the passenger strand. Although the enzyme responsible has not been identified, deep sequencing results demonstrate that Ago shRNAs subsequently acquire a short poly A 3′ tail that is then trimmed by the Poly(A) specific ribonuclease (PARN) to remove the tail and the rest of the passenger strand [123]. Compared to target matched shRNA designs, the Ago shRNA design is less potent [124], perhaps because the guide strand does not benefit from recruitment of the Dicer complex prior to loading into the RISC. Potential benefits of the Ago shRNA design include more precise processing, elimination of off-target effects from the passenger strand and activity in cell types with low levels of Dicer expression, such as monocytes [121].

## 4. RNAi Therapies for HIV-1

### 4.1. Anti-HIV-1 siRNAs

Several canonical and Dicer substrate siRNAs have been developed as potential HIV-1 therapies and many different delivery approaches have been evaluated for their systemic administration (reviewed in [125,126]). A potential advantage of anti-HIV-1 siRNAs over current therapies is that their sequences could be tailored to target a patient’s particular viral strains and provide a personalized approach to therapy. They would also open up several new targets for combination HIV-1 drug therapy. A major challenge for the development of anti-HIV-1 siRNAs is that lymphocytes, which represent the major cell-type for HIV-1 replication, are widely distributed in the body and extremely difficult to penetrate with existing siRNA delivery technologies (reviewed in [127]). To compete with current HIV-1 drug therapies, anti-HIV-1 siRNAs will need to have a long shelf life, be delivered orally and bring viral loads to undetectable levels with at least a once daily dose. While siRNAs could soon be used to treat other viral infections with limited treatment options and more easily accessible target cells, such hepatitis B virus [128], Ebola [129] and respiratory syncytial virus [130], a lot of progress in RNA delivery technologies will be needed before siRNAs can compete with small molecules for HIV-1 therapy.

### 4.2. Anti-HIV-1 shRNAs

#### 4.2.1. shRNAs Targeting Human Genes

Three large-scale siRNA screens have been conducted to identify human genes that play a role in HIV-1 replication [131,132,133]. However, only three genes (MED7, MED8 and RELA) were found in common between the studies and well characterized co-factors of HIV-1 replication, such as LEDGF/p75, were not identified in any of them [134]. In 2011, Eekels et al. tested several shRNAs targeting 30 human genes that were previously shown to contribute to HIV-1 replication [135]. From these genes they identified TRBP, ALIX and AGT6 as being most suitable for long-term inhibition of HIV-1 replication with minimal toxicity in shRNA-transduced T lymphocytic cell lines. They did not evaluate CCR5, since this gene had already been confirmed as a good target for shRNA therapy. An advantage of targeting mRNAs of cellular proteins involved in HIV-1 replication is that it would be very difficult for HIV-1 to develop resistance to the knockdown of a critical factor used for its replication. The major limitation is that targeting cellular genes can result in unpredictable side effects and a thorough examination will be required to determine whether down-regulating a particular HIV-1 co-factor can be tolerated in long-term therapy. As with drug therapies, the CCR5 entry co-receptor remains the most attractive HIV-1 co-factor for targeting by shRNAs, and a CCR5 mRNA targeting shRNA has been included in two of the three combinations that have advanced to clinical trials (Table 1).

#### 4.2.2. shRNAs Targeting HIV-1 RNA

shRNAs have been designed to target every coding and non-coding region of HIV-1 RNA and one shRNA targeting the overlapping tat/rev exon 1 reading frame has advanced to clinical trials (Table 1). The most important consideration for the design of shRNAs targeting HIV-1 RNA is that their target site is conserved across circulating HIV-1 strains. In 2006, ter Brake et al. screened HIV-1 RNA for highly conserved shRNA target sites by calculating sequence homology in 20 nucleotide windows among all complete HIV-1 sequences available in the Los Alamos HIV-1 database (170 at the time of analysis). Nineteen target regions were identified with high sequence conservation and out of 86 shRNAs, 21 were shown to have strong effects against HIV-1 production [136]. Four were efficacious in transduced T lymphocytes [137,138] and three were evaluated in a mouse model [139].

In 2007, a second genome-wide screen for highly conserved siRNA target sites in HIV-1 RNA was published by Naito et al. [140]. Similar to the ter Brake et al. study [136], they calculated sequence conservation in 21 nucleotide windows using all complete sequences available on the Los Alamos HIV-1 database (495 at the time of analysis). They identified 216 target sequences with greater than 70% conservation and selected 41 based on activity predictions from different algorithms available at the time [141,142,143]. siRNAs targeting 39 of these sequences were shown to be active against HIV-1 production; however, whether these results can be extrapolated to the development of shRNA therapies is unclear. In 2009, McIntyre et al. published a new set of sequence conservation estimates using all full-length and partial gene sequences available on the Los Alamos HIV-1 database, in addition to 150 proprietary subtype B sequences [144]. Ninety-six shRNAs were designed and ranked based on their conservation estimates and efficacy against HIV-1 production. One of the top shRNAs targeting the R region of the HIV-1 long terminal repeat (LTR) has since been evaluated in a humanized mouse model in combination with an shRNA targeting CCR5 mRNA [145].

In a screen for highly accessible target sites in HIV-1 RNA, Low et al. demonstrated that concentration dependent studies can be used to distinguish potent shRNAs from among different active inhibitors [146]. They also provided evidence that a selective 2′-hydroxyl acylation analyzed by primer extension (SHAPE)-probed RNA secondary structure for HIV-1 strain NL4-3 [147] could be used to identify single stranded regions in HIV-1 RNA that were accessible to inhibition by anti-HIV-1 shRNAs. We screened HIV-1 RNA for highly conserved ribozyme target sites and identified a sequence in the Gag coding region that was particularly accessible to inhibition by a ribozyme and could be used to design an shRNA with potency similar to the tat/rev targeting shRNA used in ongoing clinical trials [97]. In the SHAPE-probed secondary structure of HIV-1 strain NL4-3 [147], the Gag target site we identified was in a predominantly single stranded loop and we provided evidence that the accessibility of this target site to an shRNA was conserved across diverse strains [97].

In addition to being present in most circulating strains, an advantage of targeting highly conserved sequences in HIV-1 RNA is that it may be more difficult for the virus to develop resistance by acquired mutations in the shRNA target sequence, a major mechanism of viral escape from shRNAs (reviewed in [148]). HIV-1 can also escape from shRNAs by acquired mutations in sequences outside the target site that alter its accessibility, likely due to changes in RNA structure [149]. Conservation in RNA structure therefore, may be as important as sequence conservation for the design of shRNAs. Additional data on the secondary structure of HIV-1 RNA in diverse HIV-1 strains could help in the identification of target sites that are conserved in both sequence and structure. shRNAs can also be evaluated for their potency against diverse viral strains to determine whether their target site is conserved in its accessibility, and potentially in its structure [97].

## 5. Conclusions and Perspectives

HIV-1 infection can be treated with combination drug therapy. Although effective in preventing disease progression, adherence to medication, drug side effects and the potential for development of multi-drug resistant virus remain major obstacles for effective treatment in many individuals. Interventions that could lead to a temporary or permanent remission of infection, often referred to as a functional cure, could remove these obstacles. Engineering HIV-1 resistance in HSCs, the progenitors of all major HIV-1 producing cells, has the potential to functionally cure HIV-1 infection and RNAi therapeutics are among the top candidates for engineering resistance.

Although proof-of-concept for HIV-1 resistant HSC transplant was provided with the unique set of circumstances that lead to a confirmed functional cure for the Berlin patient [150], many challenges remain in identifying a broadly applicable clinical protocol to replicate these findings in other individuals. In addition to receiving an allogeneic HSC transplant from a donor with the HIV-1 resistant homozygous CCR5∆32/∆32 genotype, the Berlin patient also underwent an intense conditioning regimen including total body irradiation, immune suppression and CD3^+^ T cell depletion [151]. While this procedure could be adapted for other HIV-1 infected individuals with malignancies, the risks associated are certainly unacceptable for the general HIV-1 infected population [152]. The current approach to translate the case of the Berlin patient into an acceptable clinical protocol for all HIV-1 infected individuals is to use an autologous HSC transplant with gene-modified cells. For this approach to be successful, the gene-modified cells must efficiently engraft and repopulate the immune system. To ensure a good level of engraftment, some form of conditioning regimen will be required to reduce the number of gene unmodified cells [153] and alternative protocols are being investigated in ongoing clinical trials (Table 1).

Another avenue for the use of HIV-1 resistant cell transplant is to harvest, isolate and modify CD4^+^ T lymphocytes (Table 1), the main HIV-1 producing cell type. While the transplant of these cells would not be able to provide a complete functional cure, the procedure may be able to produce a long term remission or temporary functional cure. In addition, the toxicity and efficacy of different candidate genes could be evaluated before use in a more permanent HSC transplant.

Because HIV-1 can develop resistance to small RNAs or peptides targeting its replication, a combination of several potent genes will be needed to maintain HIV-1 restriction in transplanted CD4^+^ T cells or HSCs. Current combination clinical studies all include a single shRNA combined with one or two anti-HIV-1 proteins, peptides or other small RNAs (Table 1). While there is concern that multiple shRNAs could saturate cellular miRNA pathways, three anti-HIV-1 shRNAs have been expressed safely and in a mouse model of HSC cell transplant therapy [139] and it is feasible that a combination of carefully selected shRNAs could be used safely in humans. Newer CRISPR-Cas9 gene editing technologies have been successfully designed to target HIV-1 DNA sequences [154,155]. While their transient delivery could be used to target latent HIV-1 in reservoir cells, it is unlikely that their permanent expression in HIV-1 target cells could be tolerated in cell transplant strategies, due to potential off target effects and immunogenicity of the CRISPR gene editing enzyme.

A lot of progress has been made in the identification of effective RNAi therapeutics for HIV-1 infection and many advances in the RNAi therapeutics field have been driven by the desire to make new anti-HIV-1 therapies. Improvements in RNA delivery technologies could make siRNA therapies competitive candidates for combination anti-HIV-1 drug therapies and shRNAs targeting HIV-1 replication are sure to be part of HIV-1 resistant cell transplant approaches to functionally cure HIV-1 infection.

## Figures and Tables

**Table 1:** HIV-1 resistant cell transplant clinical trials.

Antiviral Gene(s)	Ex Vivo Manipulation	Status	Year	Reference
**Proteins and peptides**				
Dominant negative mutant HIV-1 Rev protein (Rev M10)	Gold particle transfected CD4^+^ T cells	Completed	1996	[22]
	Murine retrovirus transduced CD4^+^ T cells	Completed	1998	[23]
	Murine retrovirus transduced HSCs	Completed	2005	[27]
Dominant negative HIV-1 Rev protein	Murine retrovirus transduced syngeneic CD4^+^ T cells	Completed	2005	[28]
	Murine retrovirus transduced HSCs	Completed	2002, 2009	[29,30]
gp41 peptide, fusion inhibitor (C46)	Murine retrovirus transduced CD4^+^ T cells	Completed	2007	[31]
Zinc finger nuclease targeting the CCR5 gene (SB-728)	Transient expression in CD4^+^ T cells	NCT02225665, 01543152, 02388594	2014	[32]
	Transient expression in HSCs	NCT02500849	2016	[33]
CRISPR/Cas9 targeting the CCR5 gene	Transient expression in HSCs	NCT103164135	-	-
**RNA**				
Ribozyme targeting HIV-1 tat/vpr RNA (Rz2, OZ-1)	Murine retrovirus transduced CD4^+^ T cells	Completed	1998	[24]
	Murine retrovirus transduced syngeneic CD4^+^ T cells	Completed	2005	[34]
	Murine retrovirus transduced HSCs	Completed	1999, 2004	[25,35]
	Murine retrovirus transduced HSCs, Phase II	Completed	2009	[36]
Ribozymes targeting HIV-1 RNA	Murine retrovirus transduced HSCs	Completed	2003	[37]
Antisense RNA targeting HIV-1 env RNA (VRX496)	Lentivirus (HIV) transduced CD4^+^ T cells	Completed	2006	[38]
	Lentivirus (HIV) transduced CD4^+^ T cells	NCT00295477, 00131560	2013	[39]
RRE-decoy targeting HIV-1 Rev	Murine retrovirus transduced HSCs	Completed	1999	[26]
**Combinations**				
1. shRNA: tat/rev 2. ribozyme: CCR5 3. TAR-decoy: Tat	Lentivirus (HIV) transduced HSCs	NCT01961063, 02337985, 00569985	2010	[40]
1. shRNA: CCR5 2. gp41 peptide (C46)	Lentivirus (HIV) transduced HSCs and CD4^+^ T cells	NCT01734850, 02390297	-	-
1. shRNA: CCR5 2. TRIM5α-HRH 3. TAR decoy: Tat	Lentivirus (HIV) transduced HSCs	NCT02797470	-	-

CCR: C-C chemokine receptor; HSC: hematopoietic stem cell; shRNA: short hairpin RNA; TAR: transactivation response; TRIM: tripartite motif; HRH: human-rhesus; CRISPR: clustered regularly interspaced short palindromic repeats, -: study results not yet reported.
